# Evolution of SARS-CoV-2 virus and assessment of the effectiveness of COVID-19 vaccine

**DOI:** 10.12688/f1000research.28215.1

**Published:** 2021-01-18

**Authors:** Veljko Veljkovic, Vladimir Perovic, Isabelle Chambers, Slobodan Paessler

**Affiliations:** 1 Biomed Protection, Galveston, TX, 77550, USA; 2 Biomed Protection, Fargo, ND, 58104, USA; 3 Department of Pathology, Galveston National Laboratory, University of Texas Medical Branch, Galveston, TX, 77555, USA

**Keywords:** COVID-19, SARS-CoV-2, mutations, vaccine effectiveness

## Abstract

A safe and effective vaccine is urgently needed to bring the current SARS-CoV-2 pandemic under control. The spike protein (SP) of SARS-CoV-2 represents the principal target for most vaccines currently under development. Despite the presence of a CoV proof-reading function in viral replication, SP protein from SARS-CoV still extensively mutates, which might have an impact on current and future vaccine development. Here, we present analysis of more than 1600 SP unique variants suggesting that vaccine candidates based on the Wuhan-Hu-1 reference strain would be effective against most of currently circulated SARS-CoV-2 viruses, but that further monitoring of the evolution of this virus is important for identification of other mutations, which could affect the effectiveness of vaccines.

## Introduction

The current Coronavirus Disease 2019 (COVID-19) pandemic, caused by Severe Acute Respiratory Syndrome Coronavirus 2 (SARS-CoV-2), represents an unprecedented health threat resulting in over 1.1 million deaths globally ( www.worldometers.info/coronavirus) and significant economic damage. There is a consensus that development of an effective and safe vaccine represents the best strategy to control the COVID-19 pandemic, and consequently, this endeavor remains a top public health priority. Researchers and drug companies around the world are working hard to develop a COVID-19 vaccine, and currently 282 vaccine candidates are in clinical trials or will enter clinical investigation soon (clinicaltrial.gov). The spike glycoprotein (SP) of SARS-CoV-2 is the principal target for most these vaccine candidates. Despite the presence of a CoV proof-reading function in viral replication ^1^, SARS-CoV still extensively mutates, which might have an impact on current and future vaccine development. In the GISAID database more than 1,600 SARS-CoV-2 strains with unique combinations of mutations in SP are deposited. In order to fully understand the impact of the mutations on the SP properties (pathogenesis, virulence, transmissibility, immunogenicity), advanced laboratory studies are required. These investigations take time and are done in close collaboration between different research groups, institutions and academia. However, some important information about the biological effects of mutations could and should also be obtained by the *in silico* analysis of SP, which can be performed quickly and with a minimal experimental foreknowledge at the time of analysis.

Previously, a novel bioinformatics approach, which is based on electronic biology, was used in the assessment of the effect of mutations on the vaccine effectiveness (VE) for seasonal flu vaccines. Most recently, this approach allowed successful prediction of VE for two successive flu seasons ^2, 3^. Here, we used this bioinformatics approach for the analysis of the effect(s) of mutations in SP from SARS-CoV-2 virus on the effectiveness of the COVID-19 vaccines and/or vaccine candidates. The presented results showed that VE of vaccine candidates that are based on SP from the reference SARS-CoV-2 strain YP_009724390 remains between 76% and 94%, despite an extensive mutation rate in this important and likely protective antigen. Our analysis also suggests that further close monitoring of SP mutations might be essential in order to be able to respond to changes that may influence VE in the future.

## Methods

### Viruses

We analyzed the non-redundant subunit 1 of S proteins (SP1) from human SARS-CoV-2 viruses deposited in the GISAID ( https://www.epicov.org/epi3/cfrontend#18c7c7) from January to October, 2020, and SP1 from viruses isolated from mink that are presented in the same database. Prototype virus Wuhan-HU-1 (YP_009724390) is used in the analysis as the wild type (WT) and as the vaccine virus.

### Informational spectrum method (ISM)

The ISM is the virtual spectroscopy method for the analysis of the protein biological properties ^4^. This bioinformatics approach encompasses three basic steps: (i) the representation of the primary structure of the protein as the numerical sequence by the assignment to each amino acid of the corresponding value of the electron-ion interaction potential (EIIP), (ii) the transformation of the obtained numerical sequence into the informational spectrum (IS), and (iii) the calculation of the cross-spectrum (CS) between interacting proteins.

The EIIP is the physical parameter that determines the long-range interactions of biological molecules (interactions at distances >5Å) ^5^. This molecular descriptor is defined by the following equation ^6, 7^:

$$W=0.25\frac{Z^{*}\sin\left(1.04\pi Z^{*}\right)}{2\pi }\;(1)$$

where Z* is the average quasivalence number (AQVN):

$$Z^{*}=\frac{1}{N}\sum_{i=1}^{m}n_{i}Z_{i}\;(2)$$

where *N* is the total number of atoms, *n _i_* is the number of atoms of the i-th component, *Z _i_* is the valence number of the i-th atomic component in the molecule and m is the number of components. The EIIP values calculated according to the Equation (1) are given in Rydbergs (Ry).

The numerical sequence, representing the primary structure of protein, is transformed into the IS by the discrete Fourier transformation

$$X\left(n\right)=\sum_{m=1}^{N}x\left(m\right)e^{-i2\pi n\left(m-1\right)/N},\;n=1,2,...,N/2\;(3)$$

where *x(m)* is the *m*-th member of a given numerical series, *N* is the total number of points in this series, and *X(n)* are discrete Fourier transformation coefficients. In this way, the information defined by the sequence of amino acids is represented as the series of frequencies and their amplitudes. The frequencies in IS correspond to the distribution of the structural motifs with defined physicochemical properties determining a biological characteristics of a protein. When comparing proteins, which share the same biological or biochemical function, the ISM technique allows detection of code/frequency pairs that are specific for their common biological properties, or which correlate with their specific interactions.

### Phylogenetic analysis

**Figure 1.**
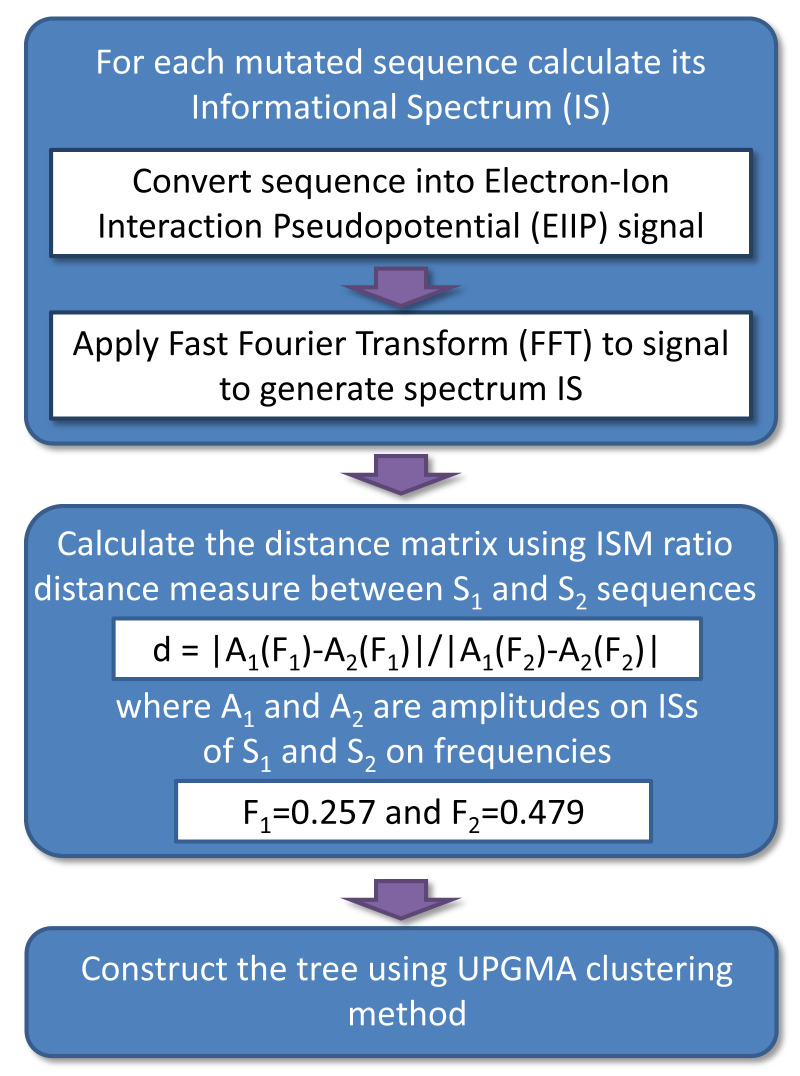
The schematic representation of the ISM-based phylogenetic algorithm ISTREE.

The algorithm of the ISM-based phylogenetic analysis, which was used for the assessment of the biological effect of mutations in SP1 proteins from SARS-CoV-2, was previously described in detail ^8^. Figure 1 provides the schematic presentation of this algorithm. Here, we used an ISM distance measure *d* defined on the ratio between specific frequencies F(0.257) and F(0.479) in IS of SP1 protein, which characterize its interaction with host factors ^9^.

## Results and discussion

**Figure 2.**
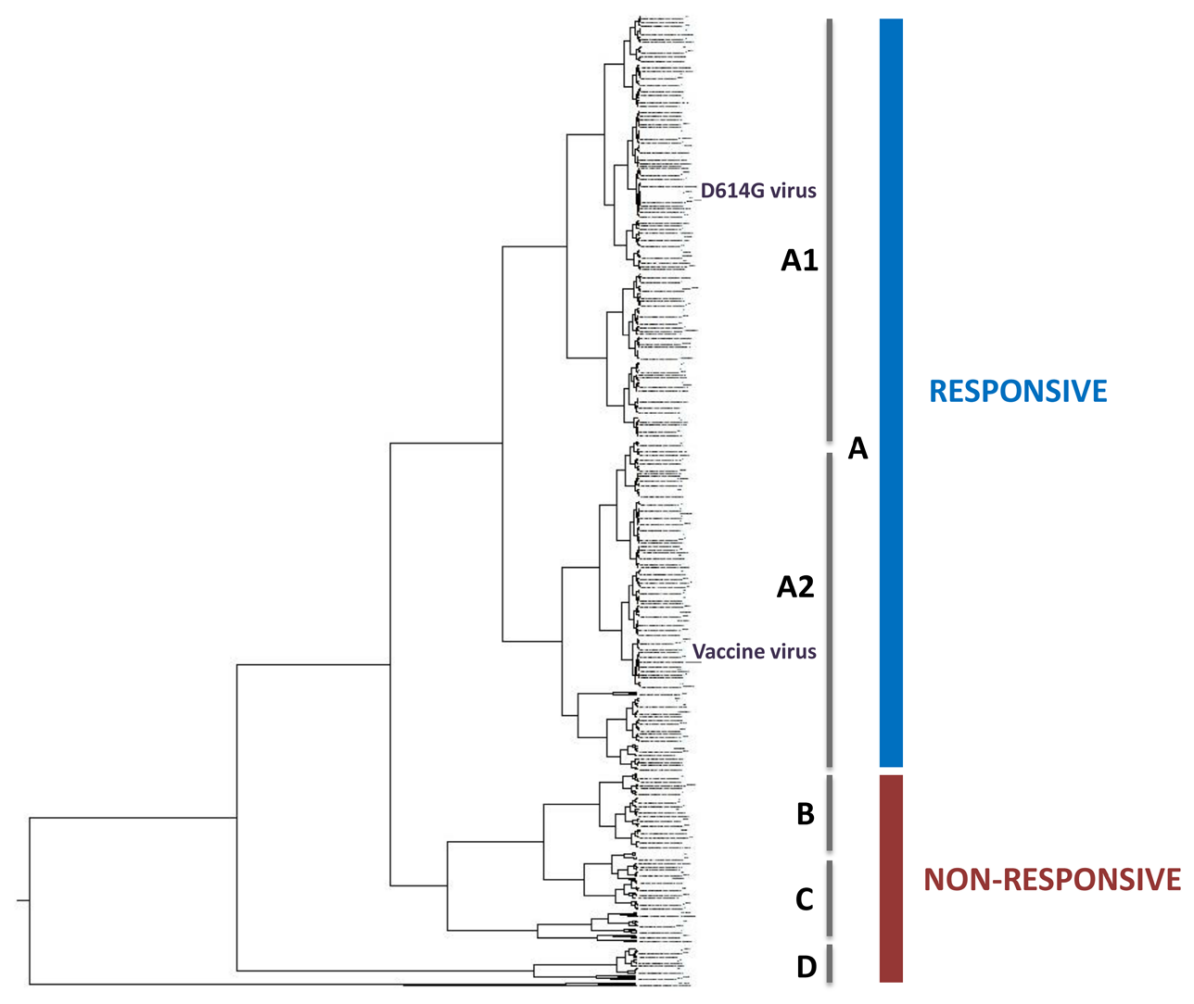
The ISM-based phylogenetic tree of the non-redundant SP1 proteins from SARS-CoV-2 viruses deposited in GISAID from January to October 2020.

Figure 2 presents the ISM-based phylogenetic tree (the high resolution tree is given in *Extended data:* Figure 1S ^10^) calculated for 1643 nonredundant SP1 proteins from SARS-CoV-2 viruses collected from January to October, 2020. Analyzed SP1s in this tree are grouped into four separated clusters. The largest cluster A, encompassing 78% of analyzed proteins, contains two sub-clusters, which includes the Wuhan-Hu-1 reference sequence for the Spike protein that is also the basis for different vaccine candidates (sub-cluster A2) and viruses with SP1 with the most abundant mutation D614G (sub-cluster A1). According to the IS concept, proteins that are grouped in the same cluster in the ISM-base phylogenetic tree have similar interacting and immunological profiles ^9, 11– 13^. This indicates that a vaccine based on SP from the Wuhan-Hu-1 reference strain will be effective against viruses that are grouped together with this virus in the sub-cluster A2. It was suggested that a single vaccine candidate based on the reference SARS-CoV-2 strain would likely match most of currently circulating variants, including the mutant D614G ^14^. Recently, it also showed that sera from D614-infected hamsters exhibit modestly higher neutralization titers against G614 virus than against D614 virus, indicating that this mutation may not reduce the ability of vaccines in clinical trials to protect against COVID-19 ^15^. This suggests that viruses which are grouped together with the mutant D614G in the sub-cluster A1 would also be responsive to the vaccine candidates that are based on the Wuhan-Hu-1 reference strain. In contrast to the cluster A, viruses that are grouped into other three small clusters B, C and D do not perfectly match the vaccine virus and might be resistant to the vaccine.

In Table 1, the distribution by months of non-redundant SP1 from potentially vaccine responsive SARS-CoV-2 is reported. These data suggest that VE of the vaccine candidates, estimated on the base of the IS parameters would be between 76% and 95%. This estimation of VE is limited with the assumption that viruses from the cluster A dominate among currently circulating SARS-CoV-2. Despite this limitation, the predicted VE is in accord with the results of the recently completed Phase-3 clinical trial of the candidate COVID-19 vaccine. This study, which enrolled 43,538 subjects, showed that the tested vaccine candidate to be more than 90% effective in preventing COVID-19 in participants.

**Table 1.** Distribution by months of the non-redundant SP1 proteins from SARS-CoV-2 viruses deposited in GISAID from January to October, 2020.

Month	Number of viruses	Vaccine responsive viruses [%]
All nonredundant SP1	1643	78
February	42	95
March	431	86
April	525	83
May	360	86
June	370	76
July	322	78
August	329	87
September	333	92
October	121	76

Although potentially vaccine non-responsive viruses in the clusters B, C and D represent only 22% of all 1,643 viruses with non-redundant SP1, these viruses could negatively affect VE. Mass vaccination, which is expected in the near future, creates an immune pressure in the population that would lead to increase in mutations in SP1 as well as in natural selection and preservation of escape mutants. Moreover, this fraction of mutant viruses that can escape vaccine protection and be efficiently transmitted could significantly increase among circulating SARS-CoV-2 once vaccination starts. For this reason, real-time monitoring of virus evolution with different methods, including the ISM-based phylogenetic tool for detection of “functional” changes in the SP1 of SARS-CoV-2 is needed.

In September 2020 in Denmark, 12 human cases of COVID-19 have been identified with SARS-CoV-2 unique variants associated with farmed minks. Viruses isolated from these patients had a combination of mutations, or changes that have not been previously observed and the implications of the identified changes in this variant are not yet well understood. Preliminary findings indicate that this particular mink-associated variant identified in both minks and the 12 human cases could negatively affect VE because mutations in these viruses moderately decreased sensitivity to neutralizing antibodies. Danish authorities have undertaken different actions to limit the further spread of this variant of the virus among mink and human populations, including culling of all farmed mink in Denmark (more than 17 million).

**Figure 3.**
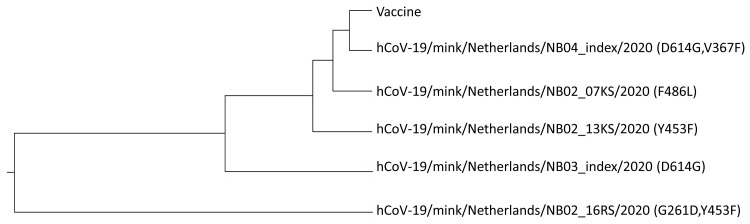
The ISM-based phylogenetic tree of the non-redundant SP1 proteins from SARS-CoV-2 isolated from minks (GISAID).

In order to assess the effect of these mutations on VE we calculated the ISM-phylogenetic tree for 5 non-redundant SP1 sequences from SARS-CoV-2 viruses from mink deposited in GISAID ( Figure 3). As presented, four of five viruses are grouped with the vaccine virus suggesting that these variants would not significantly affect VE of the COVID-19 vaccines that are based on the SP from the Wuhan-Hu-1 reference strain. The virus with the combination of mutations G261D and Y453F is out of this cluster and it could be potentially resistant to these vaccines. This suggests that further spread of this variant should be carefully monitored. Of note is that the virus with single mutation Y453F is grouped with the vaccine virus indicating that this variant will not significantly affect VE although this mutation is located in RBD. Contrary, this mutation in combination with G261D could decrease responsiveness to vaccine. This result also points out that the assessment of biological effect of mutations should be included in analysis all mutations that are present in protein.

## Conclusions

The presented analysis of SP1 proteins, performed by the electronic biology tool, suggest (i) that vaccine candidates based on the Wuhan-Hu-1 reference strain would be effective against most of currently circulated SARS-CoV-2 variants, and ii) further ISM-based monitoring of the evolution of SARS-CoV-2 is important for identification of other mutations, which could affect the effectiveness of vaccines against this virus. Therefore, the scientific community needs to be proactive in submitting genetic sequences into databases such as GSAID. For example, we have States such as Wisconsin, South and North Dakota with low mortality rate 0.8, 1.2, 1.2, respectively (GISAID accessed on November 39, 2020), but no ability to check genetic sequences of viruses from those States as none were deposited. It is very important to support genetic data collection and to share that data in real time in order to organize successful monitoring using different approaches including the one published in this paper.

## Data availability

Sequence data of the viruses were obtained from the GISAID Database. To access the database each individual user should complete the “ Registration Form For Individual Users”. This form, together with detailed instructions, are available on the website. After submission of the Registration form, the user will receive a password. There are no other restrictions for access to GISAID. Conditions of access to, and use of, the GISAID Database and Data are defined by Terms of Use.

### Extended data

Harvard Dataverse: The ISM-based phylogenetic tree of the non-redundant SP1 proteins from SARS-CoV-2 viruses deposited in GISAID from January to October 2020, https://doi.org/10.7910/DVN/JLXWWP ^10^.

This project contains the following extended data:

- Figure 1S

Data are available under the terms of the Creative Commons Zero "No rights reserved" data waiver (CC0 1.0 Public domain dedication).
