# Supplementary material for: Evolution of SARS-CoV-2 virus and assessment of the effectiveness of COVID-19 vaccine
Source: F1000Res. 2021 Jan 18;10:28. [Version 1] doi: 10.12688/f1000research.28215.1 (PMC9490287; doi:10.12688/f1000research.28215.1)
Supplement: Peer review history

# Peer review: Reviewer response for version 1

**DOI:** 10.5256/f1000research.31208.r148965

**Published:** 2022-09-20

**Authors:** Marwa O. Elgendy

**Affiliations:** 1 Beni-Suef University Hospital, Faculty of Medicine, Beni-Suef University, Beni-Suef, Egypt

Dear Editor,

 I want to inform you that this article is an exciting piece of work. The study presented the analysis of SP unique variants using the electronic biology tool whereas SP is an important target for developing most of the SARS-CoV-2 vaccines. The authors found that the vaccine candidates based on the Wuhan-Hu-1 reference strain would be effective against most of the currently circulated SARS-CoV-2 variants and that ISM-based monitoring of the evolution of SARS-CoV-2 is important for the identification of other mutations, which could affect the effectiveness of vaccines against this virus.

Is the work clearly and accurately presented and does it cite the current literature?

Yes

If applicable, is the statistical analysis and its interpretation appropriate?

Yes

Are all the source data underlying the results available to ensure full reproducibility?

Yes

Is the study design appropriate and is the work technically sound?

Yes

Are the conclusions drawn adequately supported by the results?

Yes

Are sufficient details of methods and analysis provided to allow replication by others?

Yes

Reviewer Expertise:

Clinical Pharmacy, Respiratory system diseases and COVID-19

I confirm that I have read this submission and believe that I have an appropriate level of expertise to confirm that it is of an acceptable scientific standard.

# Peer review: Reviewer response for version 1

**DOI:** 10.5256/f1000research.31208.r77630

**Published:** 2021-01-20

**Authors:** Ursula Dietrich

**Affiliations:** 1 Georg-Speyer-Haus, Institute of Tumor Biology and Experimental Therapy, Frankfurt, Germany

The article by Veljkovic *et al.* describes the application of an *in silico *method previously developed by the group to monitor the "functional" evolution of viral sequences based on the informational spectrum method (ISM). This method is based on the primary amino acid sequences of proteins, from which informational spectra are being derived that ultimately inform about protein-protein interactions. This method allows a rapid assessment of functional alterations in evolving proteins like changes in antigenicity or receptor usage and has been validated in previous studies of the Veljkovic group. A key study was the validation of mutations in the hemagglutinin sequences of H5N1 Influenza viruses predicted by ISM to alter receptor specificity in functional in vitro studies by Schmier *et al.* (2015), which should be mentioned in the introduction. ^1^

 Here this method is applied to analyze more than 1600 non-redundant sequences of the spike glycoprotein of the SARS-CoV-2 actually circulating and deposited in the GISAID database over time to derive their immunological profiles and to detect potential escape from the actual vaccine strains. The derived phylogenetic tree uncovers 2 major clusters corresponding to the Wuhan-Hu1 reference group and a derived D614G mutant covered by the actual vaccines and 3 minor clusters representing 22% of sequences, of which vaccine coverage is not known. These results nicely show how potential candidates for vaccine escape mutants can rapidly be identified by the ISM method, which can then be proven experimentally. The authors also identified by ISM a subgroup of mink SARS-CoV-2 sequences transmitted to humans in Denmark, which represent outliers compared to the actual vaccine strains and should thus be watched carefully in the future. Overall, this paper nicely underlines the value of the ISM method, which can rapidly be performed on a huge set of related sequences to identify potentially "dangerous" outliers, which may be functionally altered in terms of virulence or antigenic escape. This sets the ground to focus experimentally on these outlier strains for functional implications of the predicted mutations, thus limiting long-lasting experimental work to essential strains. As such, the manuscript potentially contributes essentially to the control of the most acute viral health problem we are actually facing. 

 The paper is generally well written, minor linguistic issues should be corrected in the manuscript.

**Minor issues: **

- *Abstract*: "....most vaccines currently in clinical applications or under development"; "Here we present computational analysis of more than...."; "effective against most of currently circulating SARS-CoV-2 viruses. However, further monitoring....".
- *Introduction*:  "...in silico analysis of SP, which can be performed much more rapidly and with minimal experimental knowledge at the time of analysis. Furthermore, in a proof of principle study, it was shown that HA mutations predicted in silico to alter avian receptor specificity of H5N1 Influenza A viruses indeed result in enhanced specificity for human receptors (Schmier *et al*., Scientific Reports 2015).
- *Results*: In Fig. 2 it should be indicated, what percentage of sequences has been assigned to the different groups (A1, A2, B, C, D). Also in Table 1, it should be indicated in an additional column, what percentage of sequences belongs to the minor groups (B, C, D) over the months to detect a potential increase over time.

Is the work clearly and accurately presented and does it cite the current literature?

Yes

If applicable, is the statistical analysis and its interpretation appropriate?

Not applicable

Are all the source data underlying the results available to ensure full reproducibility?

Yes

Is the study design appropriate and is the work technically sound?

Yes

Are the conclusions drawn adequately supported by the results?

Yes

Are sufficient details of methods and analysis provided to allow replication by others?

Yes

Reviewer Expertise:

Virology, HIV-1, Influenza Viruses, vaccine development, virus entry, antigenicity

I confirm that I have read this submission and believe that I have an appropriate level of expertise to confirm that it is of an acceptable scientific standard.

## References

[1] : In Silico Prediction and Experimental Confirmation of HA Residues Conferring Enhanced Human Receptor Specificity of H5N1 Influenza A Viruses. *Sci Rep* .2015;5: 10.1038/srep11434 11434 10.1038/srep11434 26091504PMC4473683
